# Well-Established and Traditional Use of Vegetal Extracts as an Approach to the “Deep Roots” of Cough

**DOI:** 10.3390/children11050584

**Published:** 2024-05-11

**Authors:** Luca Pecoraro, Enrico Peterle, Elisa Dalla Benetta, Michele Piazza, Grigorios Chatziparasidis, Ahmad Kantar

**Affiliations:** 1 Pediatric Unit, Department of Surgical Sciences, Dentistry, Gynecology and Pediatrics, University of Verona, 37126 Verona, Italy; 2 General Practitioner, 30100 Venezia, Italy; 3 General Practitioner, 37100 Verona, Italy; 4 Faculty of Nursing, University of Thessaly, 38221 Volos, Greece; 5 School of Physical Education, Sport Science & Dietetics, University of Thessaly, 38221 Volos, Greece; 6 Pediatric Cough and Asthma Center, Istituti Ospedalieri Bergamaschi, University and Research Hospitals, 24036 Bergamo, Italy

**Keywords:** vegetal extracts, cough, carob syrup, honey, black currant dry extracts, caraway fruit dry extracts, althea root fruit dry extracts, ginger rhizome dry extracts, ivy leaf dry extracts

## Abstract

Cough is a common presenting symptom for patients in a primary care setting and significantly impacts a patient’s quality of life. Cough involves a complex reflex arc beginning with the stimulation of sensory nerves that function as cough receptors that stimulate the cough center in the brain. This “cough center” functions to receive these impulses and produce a cough by activating efferent nervous pathways to the diaphragm and laryngeal, thoracic, and abdominal musculature. Drugs that suppress the neural activity of cough are non-specific as those treatments are not directed toward pathogenic causes such as inflammation and oxidative stress. Moreover, they block a reflex called the watchdog of the lung and have a defense mechanism. Acute respiratory infections of the upper and lower airways most commonly cause acute cough. In contrast, the most common causes of chronic cough are upper airway cough syndrome, asthma, and gastroesophageal reflux disease, all associated with an inflammatory reaction at the level of the cough receptors. The use of natural compounds or herbal drugs such as carob syrup, dry blackcurrant extract, dry extract of caraway fruit, dry extract of ginger rhizome, dry extract of marshmallow root, and dry extract of ivy leaves, to name a few, not only have anti-inflammatory and antioxidant activity, but also act as antimicrobials, bronchial muscle relaxants, and increase gastric motility and empty. For these reasons, these natural substances are widely used to control cough at its deep roots (i.e., contrasting its causes and not inhibiting the arch reflex). With this approach, the lung watchdog is not put to sleep, as with peripheral or central inhibition of the cough reflex, and by contrasting the causes, we may control cough that viruses use at self-advantage to increase transmission.

## 1. Introduction

Coughing is an essential defense mechanism of the respiratory system, allowing for removing mucus, harmful substances, and infectious agents from the airways. It is a complex and vital physiological response activated in response to various irritating stimuli. Nearly all pathologies affecting the respiratory system can cause coughing, manifesting in various forms such as dry or productive (wet), hacking, or persistent cough [1]. The cough reflex involves an intricate system of peripheral receptors, nerve pathways, and brain centers that work synergistically to generate and coordinate the cough response. Cough receptors in the airway mucosa are sensitive to irritating stimuli and harmful substances including dust, smoke, chemicals, or accumulated secretions in the respiratory tract. These receptors detect such substances and send signals to the cough center in the brainstem where they are processed and integrated [2]. The cough center, located in the medulla oblongata and the nucleus of the solitary tract, receives and integrates signals from cough receptors, orchestrating the sequence of events leading to cough reflex generation. This includes sending nerve impulses to the respiratory muscles involved in coughing including the diaphragm, intercostal, abdominal, and laryngeal muscles [3]. The phases of coughing including inspiration, compression, and expulsion result from the precise coordination of respiratory muscles and intricate neural control. During the inspiratory phase, rapid and deep inhalation fills the lungs with air, followed by the compression phase, during which the upper airways close and respiratory muscles contract suddenly, increasing intrathoracic pressure. Finally, the elevated intrathoracic pressure is released in the expulsion phase, opening the upper airways and allowing rapid airflow to expel harmful particles [4]. In addition to direct coughing, the respiratory system has defensive mechanisms such as mucus secretion and ciliary movement, which contribute to removing foreign substances and harmful particles from the airways. During a coughing episode, other associated reflexes may also be activated such as the gag reflex or the breathing reflex to increase the effectiveness of airway clearance [5]. Coughing can be a symptom of various respiratory pathologies such as viral or bacterial infections, allergies, asthma, chronic obstructive pulmonary disease (COPD), cystic fibrosis, lung cancer, and other chronic lung diseases. Under these conditions, the cough reflex can be hypersensitive, overly activated, or compromised, leading to chronic, dry, or productive cough [6].

## 2. Pharmacological Suppression of Cough

**Figure 1:**
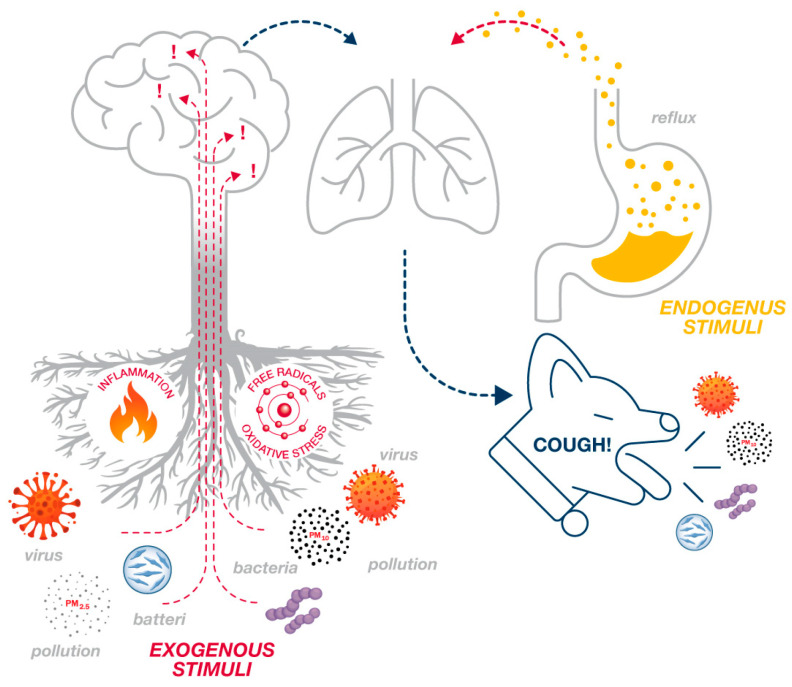
The cough is “the watchdog of the airways”; therefore, it should not be sedated, but rather, we should try to attenuate the stimuli that evoke it by intervening on the “deep roots” of the problem.

Pharmacological suppression of cough is mostly achieved with molecules that either act at the brain level or the afferent pathways of the stimulus (Figure 1).

**Figure 2:**
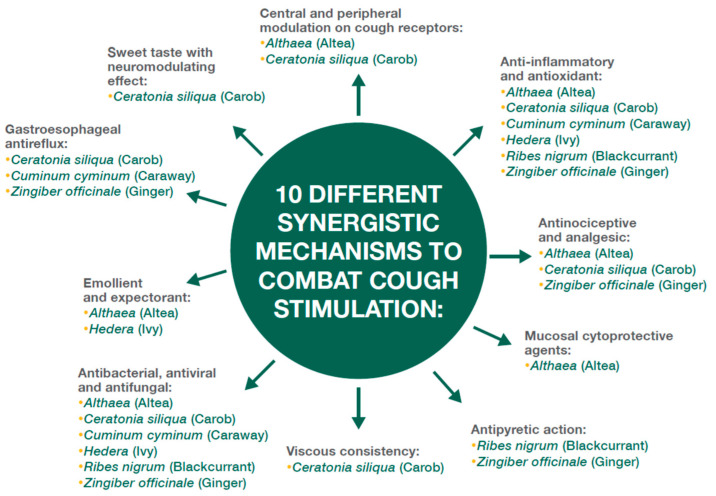
The biological approach to coughing uses natural, vegetal medicines with antimicrobial, anti-inflammatory, antioxidant, and anti-reflux effects.

This approach can be inadequate and sometimes ineffective [7,8,9] and may also have side effects [10], but above all, it acts downstream of the problem and not on the causes, namely the inflammatory response induced by the infection [11]. Since cough is commonly referred to as “the watchdog of the lung” [12], just as one would want the watchdog not to sleep during its activity, the same should be considered for the cough reflex, which should not be suppressed but potentially less stimulated for the neutralization of harmful substances. This latter goal can be achieved by using plant-based and natural substances with antiviral, anti-inflammatory, antioxidant, cytoprotective, moisturizing, soothing, and anti-reflux effects [13] such as carob syrup, black currant dry extract, caraway fruit extract, ginger rhizome extract, marshmallow root extract, and ivy leaf extract, which act on multiple factors involved in the activation of the cough reflex during viral infection (Figure 2) [14].

Furthermore, the use of dry extracts allows for standardization of the active ingredient content, that is, the complex vegetal matrix, which allows for the synergistic therapeutic effect between the various components of the plant and does not contain alcohol, which is important in both pediatric and elderly patients [15].

## 3. Properties of Various Natural Components

### 3.1. Carob Syrup (Ceratonia siliqua *L.*)

Carob syrup is obtained from carob fruits, cultivated on suitable land, whose pods are rich in carbohydrates, proteins, and minerals such as potassium, phosphorus, calcium, and polyphenols [16]. On the palate, carobs have a pleasantly sweet taste, often associated with the aroma of cocoa. Indeed, carob syrup has two essential characteristics in cough management: it is sweet and viscous. Viscous syrups formulated as cough medicines are known as “linctus” and boast a history of thousands of years, probably related to the properties of the first cough medicine: natural honey. This substance has been used as a cough medicine for thousands of years and is still very popular, although it should not be used in the first year of life due to possible contamination with spores of the bacterium Clostridium botulinum [17]. Unlike foodborne botulism, infant botulism is caused by the ingestion of spores, not by the ingestion of preformed toxin. The spores do not germinate in older children because of gastric acidity. Infants younger than 12 months have an immature immune system, a relative lack of gastric acidity, and diminished bacterial flora, which increases the risk of botulism [18]. The source of the spores is usually unknown, but some cases have been attributed to the ingestion of honey that may contain the spores of this germ.

### 3.2. Honey

Honey was the first sweetener for cough medicines, but from the fifteenth century onward, sugar cane and sugar beet began to provide a much cheaper and more readily available sweetening resource. They were predominantly used in cough preparations as inverted sugar syrup, often called artificial honey, for its sweetness and viscosity [19]. It has been proposed that the antitussive effect of sweet syrups depends on a modulating activity exerted by the sweet taste on the solitary tract nucleus in the brainstem [20]. In fact, in healthy volunteers, capsaicin-induced cough is inhibited by sweet taste but not by bitter taste [21,22], demonstrating that sweet taste may have a specific antitussive activity rather than simply being a pleasant perception for the patient. Sweet taste-induced analgesia is a physiological phenomenon with pain-relieving properties. Indeed, it is known that sweet solutions such as sucrose and glucose increase the pain threshold to various stimuli, from puncture to cold [23]. When held in the mouth, sweet solutions induce changes in the activity of endogenous opioids and the positive affective state of the brain [24]. The increase in endogenous opioid activity and affective state constitutes the biological rationale for the evidence that sweet-tasting solutions exert analgesic effects by reducing the activation of central structures involved in pain perception. Indeed, functional magnetic resonance imaging has shown reduced activation of the anterior cingulate cortex, insula, posterior parietal cortex, and thalamus when painful stimuli were evoked in association with sweet taste perception in the human brain [25]. The mechanism of action of sweet taste on cough is summarized in Table 1 [26]. However, natural honey widely varies in its composition, color, and taste, and sugars such as glucose, fructose, maltose, and sucrose represent, in variable combination, 95–99% of the dry matter of honey [27]. Moreover, widespread discomfort due to extreme weather events such as droughts, fires, and storms has become increasingly common, and these climate changes directly impact honey production and quality, as they contaminate or deplete the sources of nectar available to bees. Prolonged unusual weather patterns disrupt flowering cycles, increase the physiological need for water of bees, limit their movement patterns, decrease apiary safety, and increase the incidence of pests and diseases, all factors that have led to bee mortality with consequent loss of pollen collection and reduced honey production [28]. In addition to climate change, there is the problem of agricultural contamination with pesticides and antibiotics [29]. Bee products such as honey are widely consumed as food and medicine, and their possible contamination with pesticides, heavy metals, bacteria, and radioactive materials can pose serious health risks [30,31]. Pesticide residues cause genetic mutations and cell degradation [32], and the presence of antibiotics may exert selective pressure to develop resistant human or animal pathogens [33]. Another important aspect of honey and carob syrup is its viscosity, because a viscous medicinal formulation adheres to the oral mucosa and esophagus and induces a more prolonged taste stimulus than an aqueous medicine. Indeed, viscous syrups like honey provide a longer-lasting sweet taste than sugar water because they tend to adhere to the oral mucosa and teeth. To achieve this effect at a low cost in cough preparations, honey is replaced by high concentrations of glucose and other sugars as well as inverted sugar or glycerol. Indeed, the most common thickening and sweetening agent used in cough syrups is glycerol, also known as glycerin, which is present in 48 products and listed as the active ingredient in 17 cough medicines [34]. Glycerol is a small molecule with three carbon atoms, and its viscous nature is because each of the carbon atoms is attached to a hydroxyl group that can bind to the hydrogen atom of water, making it highly water-soluble, or bind to the hydrogen of other glycerol molecules, favoring the formation of a rather viscous molecular agglomerate [35,36]. Although the product characteristic summary often states that “glycerol has emollient properties and may potentially block sensory receptors in the respiratory tract”, no evidence has been found in the literature to support this direct effect [34]. Propylene glycol is the second most commonly used thickening agent in 20 cough medicines [34]. It has a sweet taste and useful properties as a solvent, antimicrobial, preservative, humectant, lubricant, and emollient, but it must be used purely because its contamination can have devastating effects [37].

It is hypothesized that the combination of sweet sensory stimulation associated with viscosity is responsible for at least 80% of the antitussive effect of many drugs [35]. A natural product that combines these two characteristics is carob, which also has a limiting effect on gastroesophageal reflux and contains polyphenols and flavonoids with high antioxidant capacity [38]. *Ceratonia siliqua* (carob) has an antidepressant effect mediated by dopamine and noradrenaline [39], which can contribute to controlling the cough reflex arc [40] and reduce the problem of silent aspiration [41], an event also countered by carob itself [38]. This botanical species also has antibacterial, anti-inflammatory, antinociceptive [42], and analgesic [43] effects. Countering a nociceptive stimulus has an antitussive [44] and gastroesophageal reflux-limiting effect [45]. Moreover, carob contains substances with a modest action on central and peripheral benzodiazepine receptors [46], giving this fruit both an anxiolytic and cough sedative effect [47]. Finally, this natural product can be used as a vehicle for the controlled release of other effective active ingredients [48].

### 3.3. Black Currant Dry Extract (Ribes nigrum folium)

Black currant dry extract is rich in anthocyanins and proanthocyanidins, with antiviral activity against influenza A viruses, amongst the major causes of highly contagious severe respiratory diseases interfering with virus internalization [49]. It has antioxidant and anti-inflammatory effects via a multitude of biochemical mechanisms. In the airways, it reduces eosinophil recruitment and alleviates eosinophilic-driven airway inflammation, which is particularly important in childhood asthma [50]. It reduces the duration of fever and diarrhea [51], signs frequently associated with cough in the case of influenza. It inhibits the general pro-inflammatory NF-kB system and enhances PAR-γ gene expression, supporting the adrenal cortex in cortisol production and decreasing serum hs-CRP [52]. Moreover, black currant inhibits the production of inflammatory factors suppressing pro-inflammatory M1 macrophage polarization at the cough stimuli level [53].

### 3.4. Caraway Fruit Dry Extract (Carum carvi)

Carum carvi or caraway is traditionally used for treating indigestion, expelling gas from the stomach or intestines to relieve flatulence or abdominal pain or distension, thus having a carminative effect, and for pneumonia for its antimicrobial effects [54]. It has antioxidant effects that increase superoxide dismutase, catalase, and glutathione peroxidase [54,55]. It is an anticough medicine because it promotes stomach emptying, thus reducing the risk of gastric juice microaspiration due to increased intra-abdominal pressure during coughing [56]. It is well-known that gastroesophageal reflux may occasionally cause cough, but also that cough might induce gas refluxate from the stomach in the airways, thus establishing a vicious aggravating circle.

### 3.5. Ginger Rhizome Dry Extract (Zingiber officinale Roscoe)

Ginger rhizome dry extract has been used in Ayurvedic medicine for many years to treat fever, cough, and respiratory difficulty. It exerts a powerful antitussive [57] and anti-inflammatory [58,59] action. It inhibits the production of cytokines released at the infection site by macrophages without altering their ability to present antigens to lymphocytes [60] and blocks cyclooxygenase 1 (COX-1) [61]. It has an antioxidant, immunomodulatory, analgesic [62], antipyretic, and antiemetic effect [63], with an antihistamine effect similar to that of loratadine and with fewer side effects, primarily drowsiness [64]. Ginger is considered a safe remedy [65], even in frail patients such as the elderly with COVID-19 [66], and it is also considered safe concerning its antiviral effect [67]. The monographs on natural medicinal products from Canada and Germany support using ginger as an expectorant and antitussive to relieve the symptoms of bronchitis, cough, and cold [68,69].

### 3.6. Althaea Root Dry Extract (Althaea officinalis *L.*)

The genus Althaea derives from the Greek word “althein”, meaning “to heal”. A. officinalis is used in children and infants and is considered safe during pregnancy and breastfeeding [70]. It is recommended for the symptomatic treatment of dry cough and hyperemia of the oral and pharyngeal mucosa in children over three months of age [71], with an excellent efficacy and safety profile [72]. Althaea polysaccharides promote the healing of inflamed mucous membranes and are, therefore, also used for oral rinses and gargles before swallowing [73], as recommended by the British Herbal Compendium [74]. The emollient effects of Althaea root extracts are due to their high content of polysaccharide hydrocolloids, which form a protective coating on the oral and pharyngeal mucosa, with soothing action against local inflammation [75]. It has numerous other effects: antimicrobial against bacteria and viruses, antioxidant, anti-inflammatory, immunomodulatory, and analgesic as well as promoting wound healing [76], and is also therefore used for treating lower respiratory tract infections [77]. The main polysaccharide constituent of common Althaea mucilage has a dose-dependent antitussive action comparable to that highlighted by codeine; the mechanism of action, still to be clarified, does not seem to be due to bronchodilation, but it could involve serotonin receptors [5-HT2] through a peripheral mechanism of cough reflex suppression [78,79]. Its beneficial effects are amplified by ginger and ivy [80], with an antitussive effect comparable to dextromethorphan [81].

### 3.7. Ivy Leaf Dry Extract (Hedera helix), a Herbal Medicine

For decades, the dry extract of ivy leaves has been used to treat respiratory diseases accompanied by productive cough, which should not be suppressed but facilitated in its action of removing microbial agents and pro-inflammatory cytokines. Ivy extract does not inhibit the cough reflex but increases sputum volume due to its triterpenic saponins, which have secretory properties, reduce mucous viscosity, and facilitate its expectoration [82]. Specifically, there are at least two ways in which mucus can be eliminated from the lungs by coughing: *first*, overcoming the adhesive interactions between mucus and the cell surface to detach the mucus from the airway surfaces, and/or *second*, fracturing the mucus itself (i.e., overcoming the cohesive interactions of the mucus) to eliminate the mucus in fragments [83], effects that are favored by the saponins contained in ivy. Ivy saponins have antibacterial [84], antifungal [85], and antiviral [86] properties. A study on 7034 adults treated with ivy extract for respiratory problems documented faster healing and less inappropriate antibiotic prescribing [87]. A meta-analysis of studies on using ivy extract in subjects with acute cough showed a significant reduction in symptoms from the second day of treatment with side effects comparable to those observed in patients treated with the placebo [88]. Ivy also inhibits the internalization of β2-receptors [89], improving respiratory function in asthmatic children treated with budesonide [90] and stimulating surfactant production with mucolytic effect by type II alveolar cells [91], consequently reducing the symptoms in adults with chronic bronchitis [92]. This principle reduces the release of interleukin 6 [93] and NF-kB activation [94], with a consequent anti-inflammatory effect useful in the adjunctive treatment of cough in both acute [95] and chronic [96] respiratory diseases. The tolerance and safety of various preparations containing ivy leaf extracts have been tested and confirmed in several studies [97], with a low incidence of adverse events in children of all ages including those under 1 year old [98]. The volume of randomized, controlled, and double-blind studies (RCTs) related to Hedera helix folium is such as to qualify this standardized herbal extract as WEU (well-established use), safe and effective akin to a class A drug [99]. Among all natural products for productive cough, ivy is unrivalled and constitutes an excellent medical prescription, a true herbal medicine. The European Medicines Agency (EMA), by classifying it as WEU, guarantees that the scientific level of proven safety and the efficacy of the medicinal plant has passed rigorous controls exactly like any other conventional drug to which a marketing authorization (MA) is granted [100]. For each evidence-based plant, the EMA compiles a monograph that encapsulates the entire body of studies, periodically updated, which is a “Final assessment” [101], a “Final list of references supporting the assessment” [102], a more concise monograph that functions like a leaflet with the composition, indications, dosage, contraindications, adverse effects called the “Final European Union herbal monograph” [102], and a “Summary for the public” [103], a list of questions and answers for ordinary people who want to know more about the medicine they are taking.

## 4. Other Herbal Medicines

Thyme (*Thymus vulgaris* L.) is a widely used aromatic plant in traditional medicine to treat various diseases including diarrhea, fever, cough, irritation, skin diseases, rheumatism, respiratory disorders, influenza, and digestive problems. It contains various classes of secondary metabolites such as terpenoids, alkaloids, flavonoids, tannins, coumarins, quinones, carotenoids, and steroids with potent antibacterial, antifungal, antipyretic, antinociceptive, antioxidant, and anti-inflammatory effects [104,105,106]. It has a proven antitussive effect with double-blind studies in children and adults, especially combined with ivy extract [107,108]. These preparations reduce the frequency and severity of coughing and facilitate mucus lysis and expectoration, improving the quality of life [109]. Adverse events reported were mild, like those found in the placebo groups [110]. The most important component responsible for thyme activity is thymol contained in thyme volatile oil, which, combined with primrose or other natural substances, effectively controls coughing in subjects with upper respiratory tract infections [111,112]. In children with acute asthma, a thyme-based syrup administered every eight hours significantly reduced coughing and improved respiratory function compared to the control group, suggesting its potential use as an adjunct in asthma exacerbation management [113]. In patients with COVID-19, inhalation of thyme oil was effective in relieving symptoms such as shortness of breath, dizziness, secretion, diarrhea, weakness, loss of appetite, coughing, headache, and muscle and joint pain compared to the control group [114]. The root extract of primula (*Primula veris* L.) has also been used in treating cough, essentially in combination with thyme and ivy [115,116,117], and is classified as WEU [116].

## 5. Other Traditionally Used Extracts (TUEs)

For TUEs, these are defined as herbal medicines used in therapy for at least 30 years worldwide, with at least 15 years within the European Union. Safety is guaranteed with the marketing authorization (MA) mark, and quality is demonstrated, but not by robust studies like WEU herbal medicines. They may be non-double-blind, non-randomized, or have a non-significant sample size. The EMA considers that a drug used without problems for decades with precise indications can be used with confidence: why deprive citizens of benefits recognized for a long time just because large sums of money could not be invested in research? The EMA carried out the enormous job of acquisition, evaluation, updating, and publication, and all this is made available free of charge on the Internet: evidence-based herbal therapy exists. Herbal extracts traditionally used in the management of patients with cough are *Pelargonium sidoides* root [118,119,120], oregano [121], and peppermint (*Mentha* × *piperita*) essential oil [122], which is curiously a TUE for cough and cold while being WEU for gastrointestinal spasms, flatulence, and abdominal pain, especially in irritable bowel syndrome [123]. Grindelia (*Grindelia inuloides*) [124,125], Icelandic lichen (*Cetraria islandica*), is an extraordinary demulcent for dry cough [126,127]. Licorice (*Glycyrrhiza violacea*), better known as a digestive aid, is also effective in cough, not only from gastroesophageal reflux, but also from colds [128,129]. *Matricaria recutita* [130], whose recognized properties range from minor intestinal disorders to oral ulcers, perineal inflammation, and sunburn, is considered a remedy for cough [131]. Even eucalyptus (*Eucalyptus obliqua*) essential oil, besides having indications of muscle spasms like rosemary essential oil, has been proposed for managing cough [132,133] and nasal congestion [134]. However, essential oils, if accidentally ingested by children, can also have significant side effects such as seizures [135], and theoretically, inhalation could induce lipoid pneumonia [136]. In any case, the EMA monograph indicates its use from age 12, specifying that it is indicated in adolescents, adults, and the elderly. One cannot go wrong if one follows exactly what the Herbal Medicinal Products Committee (HMPC) team of the EMA [99,100,101,102] reports.

## 6. Conclusions

Cough represents an important defense mechanism of the airways since it allows the removal of phlegm, harmful substances, and infectious agents from the respiratory tract. The appropriate management of this symptom cannot ignore an accurate diagnosis, considering possible underlying causes and triggering factors. However, it is important to consider that attenuating the factors responsible for the onset of cough such as infection, inflammation, oxidative stress, and aspiration of gastroduodenal secretions represents a biologically more plausible approach than simply suppressing the cough reflex at a peripheral or central level.

It is particularly important to use substances with synergistic effects, proven safety, and efficacy in extreme stages of life such as infancy and old age. This targeted approach can help reduce the risk of adverse effects and toxicity associated with the use of antitussive drugs. Since antitussive agents suffer from the lack of large trials establishing their clinical efficiency and pharmacodynamic properties, it is always essential to consult the monographs of the European Medicines Agency (EMA) to learn the specific indications, dosage, duration of use, and the contraindications and adverse effects associated with each drug. It is important to remember that the term “natural” does not necessarily mean “harmless”; therefore, it is crucial to carefully evaluate the safety and efficacy of any treatment before using it, even if based on natural substances. Furthermore, the quality of the products on the market depends on the extent to which the supply chain is respected, in other words, cultivation > harvest > drying and storage > extraction > standardization > titration > product formulation.

## Figures and Tables

**Table 1:** Biological connection between sweet taste and cough reflex.

- Sweetening substances may have emollient properties.
- Sweetening substances trigger salivation and can change the composition of saliva.
- Oral perception of sweet taste stimulates swallowing. It reduces coughing.
- Sweet taste influences reflexes related to breathing. Specifically, sugar on the tongue can inhibit hiccups, which represent an expression of respiratory reflexes.
- Oral stimulation provokes saliva production, which results in lining the throat; it is useful in soothing dry cough.
- Sweet substances can improve the rheological properties of mucus.
- Sweet substances also have analgesic effects by modulating the release of endogenous opioids.
- The antitussive and analgesic properties of opioids are related.
- Endogenous opioid release represents one of the mechanisms with which sweet taste inhibits the cough reflex. Specifically, it is related to the anatomical contiguity of the brain mechanisms involved in the cough reflex that processes taste signals. All of these primary afferents terminate in the brainstem.

## Data Availability

Not applicable.
